# Spirit of the Foreign Periodicals

**Published:** 1834-10-01

**Authors:** 


### Spirit of the Foreign Periodicals.

### On the Operation of Staphyloraphy.

Although the congenital fissure of the palate does not necessarily affect the health of the individual, it is a malady which causes him much distress, and often destroys the comfort of his existence. Every act of swallowing is a punishment; for the food, especially if fluid, is apt to pass through the cleft into the nasal passages, and be rejected by the nose; and the speech is usually so indistinct, that the unfortunate patient can with difficulty make himself understood.

It is, therefore, surprising that surgeons had never made any attempts to remedy this defect, until MM. Graefe and Roux, nearly about the same time, proposed and practised the operation which has been called staphyloraphy. It must be confessed that the operation is one of very considerable difficulty and uncertainty, in consequence of the peculiar susceptibility and disposition of the parts involved. All the manipulations are performed within a contracted cavity, the walls of which, on the slightest irritation, are thrown into irregular and involuntary movements, accompanied with irrepressible efforts of vomiting and cough. The only method of overcoming these, is by endeavouring to accustom the throat, step by step, to the contact of foreign bodies, Even when this has been accomplished, the surgeon may find himself much embarrassed in his attempts to make the section of the cleft palate neat, regular, or uniform, and to pass the needles and ligatures through the two sides at the wished-for points, so as to approximate them smoothly and equably. Any feasible proposal to facilitate these desirable ends must be received most gratefully by whoever undertakes the delicate operation of staphyloraphy. M. Berard, of Paris, suggests the following hints, the adoption of which, he believes, contributed essentially to his success in one case. The operation, he says, consists of three stages, viz. the passing of the threads, the paring of the edges, and the tying of the ligatures. The points of the suture, on either side, ought to be exactly opposite to each other; the spaces between them must be equal, or nearly so, and sufficient thickness of substance must be included between any two opposite sutures, to prevent the ulceration of the interstitial parts before the adhesive union has taken place. The contrary error, that of including too great a thickness of substance, may, indeed be committed, and its effects aie nearly quite as injurious, causing a most painful dragging of the velum and soft palate, and inducing a troublesome inflammation in these parts. It is, however, to be well kept in mind, that the failure of staphyloraphy is more often attributable to the former than to the latter of these errors.

M. B. strongly recommends that the sutures be made progressively from before backwards, and not in the opposite direction, as some surgeons have proposed and practised. In the second stage or period of the operation, the section ought to commence at the upper border of the velum; for this curtain, being fixed by its adherent edge to the point of the osseous palate, may be kept stretched by the pressure of the bistoury alone: some surgeons prefer scissors to a scalpel for paring the edges—it is, however, to be observed that, with the former, we are more apt to cut the threads than with the latter. When the incisions have been duly made, the next step is to approximate the edges, and to retain them in apposition by tying the ligatures. The most convenient of all "serre-nœuds" are the fore-fingers of the operator, which may generally be conveyed to the requisite depth; in some cases, indeed, considerable difficulty is experienced at this step of the operation, in consequence of the nausea and efforts to retch whenever the fingers touch the palate or velum; also from the surgeon not being able to see distinctly the parts on which he is operating. M. Guyot has recently invented an ingenious instrument, by means of which he thinks that the surgeon will be enabled to tie the ligatures, without the introduction of the fingers into the mouth. Even after the operation has been satisfactorily concluded, the anxiety of the surgeon is not over, for he knows well the extreme difficulty of keeping the parts quiet and motionless; and, without this favourable condition, he cannot expect a serviceable union. Every effort of swallowing is necessarily attended with some movements of the palate; and although we may endeavour to nourish the system for a few days with beef-tea enemata, and so forth, the thirst of the patient forces him to be taking every now and then a mouthful of drink, and even sometimes the urgency of his hunger is so intolerable, that he cannot abstain from wishing for food by the mouth.

It has been proposed to introduce food into the stomach by means of an elastic tube, conveyed down the œsophagus; if we intend adopting this method, it will be prudent to accustom the parts to the contact of the tube for several days previous to the operation. It may be useful to detail briefly the history of the case in which M. Berard performed the operation with success. The patient was a man, 27 years of age—he had the scar of the operation for hare-lip—his voice was hoarse, indistinct, and his speech very inarticulate. Upon examining the throat, the velum palati was found to be cleft in its middle—the fissure extended from the uvula to within two or three lines of its adherence to the palate bones; it was about an inch and a quarter in length, and had a triangular shape, the apex of the triangle being uppermost.

The palate itself, the soft as well as the hard, was quite entire. When the parts were in a state of repose, the breadth of the fissure was not more than three or four lines, but no sooner were they thrown into action, than the two halves were immediately drawn so much asunder and upwards, that they could scarcely be perceived. For several days, M. Berard endeavoured to accustom the parts to the contact of foreign bodies, by repeatedly introducing his fingers into the mouth, and touching the parts which were to be the seat of the operation. Three sutures were employed, and great care was employed to insert each of them at about three lines' distance from the loose edges of the fissure. Fortunately, this patient was able to abstain from swallowing any food for five days, nourishing injections being exhibited two or three times daily. The ligatures were removed on the sixth day; by this time, the lower half of the fissure was well cicatrized—the upper portion also adhered, but between these there was a gap, about half an inch long and a quarter broad. The edges of this gap were touched every second or third day with nitrate of silver: they gradually became approximated, and, finally, were firmly agglutinated; but not until two months from the date of the operation. The deglutition and speech of this patient have become much more easy and distinct than they were formerly.—*Archives Générales.*

---

### On the Curative Effects of Abstinence, and of a very Rigid Diet, in certain Diseases.

The Sangrado treatment has been several times tried and recommended in Italy, France, England, and Sweden, and now our German brethren have adopted it, and are extolling its salutiferous blessings. Rust assures us that he employs it extensively at the Charité Hospital of Berlin, with great good effects; and his disciple, M. Rollfs, has communicated the history of a case, the cure of which was altogether attributable to a "diète méthodique!" The case was one of spinal deformity, accompanied with paralysis of the lower limbs, and occurring in a lad 14 years of age. There was also caries of the ischium, and a fistula in the perineum. Confinement to the horizontal posture for a length of time, and the use of various remedies, had been tried, without any good effects. Issues had been kept open for several months; but notwithstanding their use, paraplegia had increased so much, that the patient could with difficulty lift either of his legs from the bed.

The "diéte méthodique" was then commenced, and the young patient voluntarily submitted to its exactions with great fortitude. One ounce of food was withdrawn each day—no animal meat nor bread was allowed. In the course of three weeks, the whole quantity of food taken in 24 hours was only eight ounces of fresh vegetables, two small cups of milk, and half a pound of grapes. The pulse had fallen from 80 to 48 beats. After other six days, it was found that he had considerably more use of his limbs, and that not only could he raise them, but also that he could stand erect for a few minutes on his feet; nevertheless, they had become considerably emaciated. The hunger was now so ravenous, that he felt almost inclined to catch and devour the flies which settled on his clothes. From this period, the amelioration of all the symptoms was very rapid and striking, for not only could he walk about his chamber, but soon he was able to go out, and in the course of a few months he became an apprentice to a baker. The perineal fistula had quite healed.

*Remarks.* It is not stated what appearances the spine presented after the cure had been effected; but our readers will doubtless agree with us in believing, that there had, in all probability, never been any considerable disease of the bones, and that the paraplegia might be owing to a slight compression of the spinal cord, from the existence of a serous or other fluid within the vertebral canal. The rapidity of the cure is inconsistent with the presumption of any serious mischief of the osseous structure. One of the most important physiological effects, says M. R. of the "diète" is, that the patient draws a portion of his nourishment from his own flesh and blood, and thus the universal interstitial absorption goes on increasing, just in proportion as the absorption from the surface of the intestines decreases. The superfluous blood is removed, the fat of the body is taken up, and every organ supplies its quota of nourishment to the support of the whole. Although a general emaciation is the consequence of all this, and, although the organs of digestion be comparatively inactive, and the circulation become languid, it is observed that the functions of the nervous system are not depressed in proportion, and that very often they even seem to be exalted. The diseases in which the "diète" is most useful, are threatened miscarriage, hæmorrhages, diarrhœa, and ephidrosis. It is also highly efficacious, we are told, in arresting seminal pollutions, especially if the patient, at the same time, engages in some serious pursuits during the day, and sleeps on a hard bed at night! Syphilis and many cutaneous diseases are often singularly benefited, and sometimes quite banished, by the penance of fasting, Gonorrhœa, too, may very generally be got rid of, in the course of one week, by restricting the patient to the use of mere barley water, and an occasional dose of the powder of cubebs. Some physicians have recommended that small doses of ipecacuan, or of tartar-emetic, be given frequently, with the view of exciting nausea, and thus suspending the desire for food; but Dr. R. assures us that this practice cannot supersede the one which he recommends; for that, in order to be of service, the feeling of hunger must be experienced, and that it is this very feeling which is often so extraordinarily efficacious in curing disease, by the state of general excitement which it induces. In conclusion, adds our starving author, it is necessary to animate and support the courage of our patients by painting, in the most glowing colours, the hope of a speedy cure, and by appealing to their honour and fortitude to assist us in the noble object they, as well as ourselves, have in view!! —*Archives Générales.*

We have great doubts that the praises of the "diète méthodique" will ever be sung, or at least will ever be listened to, with so warm an enthusiasm on this side of the Channel, as in Germany or France. It has the mighty disadvantage of being in direct antagonism (to use a new medical word) with all the inbred notion of our countrymen.—Rev.

---

### Operation of Pelviotomy. By Dr. Galbiati.

A rachitic female, about thirty years of age, was admitted into the Hospital of Incurables at Naples, pregnant with her first child, and near the end of her gestation. She was exceedingly deformed, very low in stature, and all the limbs, especially the lower ones, were bent into the most irregular shapes. On examination of the pelvis, it was found that the sacro-pubic diameter did not exceed one inch and a quarter. A consultation was held, to determine on the practice to be followed. Signor G. proposed the operation of pelviotomy, modified in the following respects from the original Sigaultian method: the two ossa pubis to be divided with strong curved scissors or nippers, at about three fingers' breadth from the symphysis; then the ossa ischii, at the posterior part, to be similarly divided; and, lastly, the symphysis pubis to be cut across and separated laterally. "By means of this quintuple section," we are told, "sufficient space may be obtained for the easy expulsion of a fœtus from a pelvis so deformed, that the Cæsarian operation would be deemed quite necessary."

The following are the steps of the operation, as performed by Sig. Galbiati. An incision was made through the soft parts over the right os pubis, and over the region of the posterior, or lower portion of the ischium; these bones were then divided across with pliers—a manœuvre not very easily done, but which, after some delay, was satisfactorily accomplished; a small intermuscular artery required a ligature; the pudic and obturator vessels were quite safe. The next step was to divide the symphysis pubis;—this being accomplished, Signor G. introduced the thumb and fore-finger of his right hand into the vagina, ruptured the membranes of the fœtus, and discharged a large quantity of the inclosed waters. The operation had by this time lasted for an hour and a quarter; and as uterine pains began to come on, the wounds were dressed, and the patient put to bed, and a large opiate was administered. The patient experienced much pain during the night, and occasionally she was delirious. On the following day, the sacro-pubic diameter of the pelvis was found to have stretched at least a third of an inch since the operation; the os uteri had become dilated, and the head of the fœtus had advanced somewhat, squeezed like a wedge between the ossa pubis, and the vast protuberance of the sacrum.

The second day after the operation, it was a question among the surgeons, whether the section of the bones on the left side should now be performed; and it was urged in favour of this step, that sufficient space might be thereby obtained for the introduction of the forceps, and the mechanical extraction of the child, of whose death there were now well-grounded suspicions. The condition of the patient was, indeed, far from being favorable; nevertheless Sig. Galbiati resolved to complete the operation, as he had proposed at first. Having, therefore, cut across the rami of the pubis and ischium on the left side, as he had done the day before on the right, he then introduced the forceps, and extracted, but not without considerable difficulty, a well-formed male fœtus, whose appearance indicated that life had ceased for about 24 hours. A belt was applied round the pelvis, and warm fomentations kept to the abdomen. On the morning after the second operation, the patient was found to be in a highly irritable and nervous state; the symptoms became gradually more alarming, the pulse fell, the body became cold, and the poor woman passed "a miglior vita" on the morning of the fourth day after the first operation, and thirty hours after the second.

*Dissection.* The three first wounds were in a sloughy gangrenous state, bedewed with sanies; the other two wounds were dry. On dividing the abdominal parietes, all the viscera appeared healthy; the uterus and its appendages presented no morbid signs; when cut through, its internal surface was, in every respect, such as might have been expected after a recent delivery. The sacro-iliac synchondroses were in a normal state; the ossa pubis and ischii on the right side were found to have been smoothly cut in their perimeters, or surfaces, and broken through rather irregularly in their substance; but none of the spicula or fragments had injured the surrounding soft parts; the bones on the left side had been more smoothly divided throughout.—The lowest lumbar vertebræ and the sacrum were greatly enlarged, and projected forwards, like a huge round mass, into the cavity of the pelvis, so that the conjugate diameter was exceedingly small.—*Giornale di Chirurgia, Napoli.*

Such is a brief history of this remarkable case;—remarkable only for the unjustifiable and barbarous boldness of the medical attendants. Really, some of our continental brethren in Italy, and, we may add, in Germany also, seem to regard the body of a parturient woman as a block of wood, on which they may cut, and carve, and chisel, with the utmost coolness. The narrator of the preceding case seems to have been quite puzzled how to account for the death of the patient! He attributes it to "a nervous affection, the result of an imagination over-excited by all the circumstances which preceded and followed the operation!!" He speaks somewhat more like a rational being, when he advises that the operation be completed at once; and that it was highly reprehensible to allow two days to intervene, before the section of the pelvic bones on the left side was performed. But the operation, however modified, ought to be denounced as murder.—Rev.

### Removal of Calculi from the Perineum.

A young boy, admitted into the hospital at Naples, presented on examination a prominent swelling of the scrotum; several hard bodies, like calculi, were felt under the integuments. Professor de Horatiis made an incision over the part, and extracted three calculi, of the size of coffee-beans, and a quantity of gravelly matter—the wound speedily healed. It is to be observed, that this boy had previously suffered severe pains in the region of the kidneys; the calculi were therefore probably of renal origin, and being driven along by the current of the urine, had become impacted in the urethra, through the parietes of which they had forced their way.—*Ib.*

### Disease of the Cerebellum.—Curious Disturbance of the Muscular Powers.

A middle-aged man was lately admitted into the Hôtel Dieu. The symptoms were feverishness, great debility, confusion of head, and inability to use his limbs, at least freely. There was no abdominal or pectoral distress. When he was put to bed, it was observed that the head was in a state of continual rotation, even when resting upon the pillow;—it was not bent forwards or backwards, but only rolled about from side to side: the muscles of the face were occasionally convulsed, but the mouth was not distorted, nor was the tongue drawn either to the right or left side, although constantly moving with a tremulous agitation. Both forearms exhibited a prolonged convulsive movement along their radial sides, so that the thumbs and forefingers were kept bent; the soles of the feet were turned inwards and upwards; the tendons of the tibiales autici muscles were very prominent, and permanently stiff. The patient seemed to have a complete control over the flexors and extensors of the head; when assisted, he could raise himself up and sit in bed. The movements of the thoracic and abdominal muscles were healthy. The voluntary movements of the extremities were very imperfect; he could not raise his left arm at all to his head, and his right one only with considerable difficulty. The common sensibility was but little affected in any part; the intellect was confused; he could not answer many questions successively. In the course of a few days all the unfavourable symptoms were aggravated; the trembling oscillation of the head and tongue was incessant; the trunk and limbs lay nearly still and motionless; the forearms were laid across the abdomen, the wrists were bent, and the thumbs drawn into the hollow of the hand, and almost constantly trembling. Every now and then there was a paroxysm of apparently epileptic convulsions, and the patient seemed to recover his sensibility and consciousness for a time, but he soon relapsed into his former stupor. On dissection, the attention of the physician was directed in a special manner to the encephalic contents; and it was found that a tumor, of the size of a small walnut, had formed on the tuber annulare, and adhered to the outer surface of the cerebellum. This tumor was formed of nacreous particles and layers, and belonged to that class of morbid deposits described by M. Cruveilhier as consisting of stearine and cholesterine;—they appear to be quite inorganic, presenting no traces of vessels, or of cellular tissue—*Archives Générales.*

---

### Hypertrophy of the Muscular Coat of the Stomach.

This pathological phenomenon has hitherto been almost quite overlooked by anatomists. Neither Morgagni, Haller, nor Baillie allude to it. Meckel, in his Manual, states, in a general way, that in great eaters the muscular coat of the stomach is often thicker than is usual. M. Louis has indeed detailed two well-marked instances of this hypertrophy which he met with; in both, the pyloric orifice was affected with scirrhus; and he was thereby led to regard the former change as the result of the latter. A woman, 49 years of age, of a spare habit of body, had long suffered from mental anxiety, which she vainly endeavoured to subdue by immoderate indulgence in the pleasures of the table;—in course of time the appetite became quite voracious. Her chief malady was a difficulty of breathing, which returned periodically every evening, and was accompanied with the sensation as if a heavy ball was rising from the lower part of the belly to the region of the heart, and there stopped, and obstructed the respiration. These symptoms lasted generally for about six or eight minutes, and then gradually subsided. Each attack became more severe; and at length they sometimes induced delirium. A general emaciation supervened, although the bulimia was not diminished. All the remedies tried to relieve her failed, and she died in the course of a few months from marasmus.

*Autopsy.* Thoracic organs healthy; omentum almost entirely awanting; liver, gall-bladder, and spleen natural. The stomach was puckered, and evidently much thickened, especially towards the pyloric orifice, and along the great curvature; its blood-vessels were empty; the mucous membrane, coated with a viscid deposit, was thin, transparent, but not softened; its surface was elevated into bold irregular projections, by the subjacent bundles of muscular fibres, extending from the great cul-de-sac towards the pylorus; between these projections were hollows, or depressions; and thus this stomach presented an appearance not unlike to that of the inner surface of the heart. The thickness of some of the muscular fasciculi was at least half an inch. The rest of the alimentary tube was normal. —*Hufeland's Journ. der Pract. Heilk.*

---

### Inflammation of the Membranes of the Ovum.

Madame ———, 18 years of age, in the fourth month of her first pregnancy, applied to Dr. Ollivier, in consequence of general indisposition, and of a reddish coloured discharge from the vagina.—The abdomen was not at first tender nor swollen, but severe pain was felt in the region of the kidneys. The movements of the child had not yet been felt. The uterus could be felt distinctly with the hand placed on the hypogastrium; and if firm pressure was made, a sharp pain was produced. Gently soothing means and absolute rest were enjoined, and after a few days the patient seemed to be relieved, when suddenly the abdomen became enormously distended, and a fluctuation was distinctly perceptible. A sense of uneasiness and weight in the hypogastrium was complained of. Dr. O. was not at all surprized at this change in the symptoms, as he had, from the commencement, regarded the case as one of inflammation of the membranes of the ovum; and he therefore concluded, when the sudden swelling of the abdomen supervened, that the inflammatory process had terminated in effusion. The treatment which he adopted was of the simplest kind, as the general health of his patient was now moderately good. The coloured discharge became less and less, all tenderness of the abdomen ceased, and the movements of the child began to be felt. The pregnancy went on favorably to its full period, and the lady was safely delivered. Dr. O. was then able to verify the accuracy of his prognosis.

It may be noticed, that during the labour, and before the head was protruded externally, there had escaped suddenly a tumor or pouch as large as a man's fist, formed apparently by the fœtal membranes, but of the thickness and consistence of softened parchment.—As labour advanced this pouch burst, and discharged the amniotic fluid.

On examination of the after-birth and membranes, he found that these last were, for about one-third of their extent, considerably thickened, opaque, and of a whitish colour, villous on their internal surface, and, in short, altogether like to parchment which had been long steeped in water. All the thickened portion was traversed by minute vessels, and these were more distinct as they approached the placenta.—*Arch. Générales.*

---

### Case of Fatal Opisthotonos, to which are appended some Physiological Remarks.

Dr. Bellingeri, with whose work on the functions of the nervous system our readers are made acquainted in a preceding part of the present number, is the narrator of the case. A country girl, thirteen years of age, whose health had been very generally good, in spite of a considerable bronchocele, began about the middle of last year to experience a pain in the left shoulder, which extended towards the neck, and was especially severe over the larynx; the neck became stiff, and occasionally she complained of pain in the occiput, and difficulty in moving the lower jaw.—These symptoms she attributed to having slept in a damp chamber. Although troublesome, they did not seem to disturb her general health, as she eat and slept well, and was able to go about as usual. It deserves, however, to be noticed, that the act of swallowing was not quite easy;—she felt as if the food was partially obstructed in its descent. In the course of a day or two, she experienced sharp pains along the whole extent of the spine, and a sense of dragging in the lower extremities. Two days after, the lower extremities became rigidly drawn backwards; and in other 24 hours, the head, neck, and trunk were similarly affected. The sharp, darting pains in the occiput and along the spine were as severe as hitherto. Venesection and oily purgatives were employed, and leeches were applied to the back. For five days, the tetanic symptoms continued in all their severity; and so powerful was the opisthotonos, that the body of this poor sufferer formed a complete arch backwards. The rigidity of the spasm was strongest in the lower extremities;—so stiffly were they drawn backwards, that they could not be brought into a straight direction, even when considerable force was used. The power over the upper extremities was not completely lost, the patient being able to move them about in some directions; but the movements were limited and also painful. The pupils were observed to be constantly contracted, and seemed to be little or not at all affected by light: the sense of vision and the hearing were not however disturbed. The alæ nasi and the upper lip were drawn upwards, and the patient could only imperfectly close the mouth: indeed every muscle of the face seemed to be more or less affected with spasm. In no part was the common sensibility affected. The pulse was contracted, and beat about 104 times in the minute. The abdomen was so stretched that its surface was as flat as a board; for four days no alvine evacuations had been obtained; and it was found that considerable spasm of the sphincter ani and of the rectum existed, when attempts were made to throw up enemata. The urine was voided voluntarily and at intervals; but she had not the power of retaining it; for whenever the desire came on, she was obliged to yield at once to it. The heat of the body was rather higher than in health; and the skin was always gently moist.

Dr. B. saw her for the first time on the ninth day after the commencement of the disease, and found the symptoms then as we have described. He tried the effects of bleeding from the jugular vein, in consequence of the patient complaining of a distressing constriction in the throat. After about nine ounces had flown, a sense of suffocation came on, and the face became livid; the bleeding was immediately stopped, and in the course of a few minutes the breathing became more easy; but again the paroxysm returned with more alarming severity, and the patient died, apparently asphyxiated, from a spasmodic closure of the rima glottidis. A short time after death the left arm was drawn backwards. For twelve hours the rigidity and distortion of the head, neck, trunk, and lower extremities continued, although the body was then quite cold; in other twelve hours these appearances had ceased, and the joints were pliant.

*Dissection.* On the posterior surface of the spinal cord, between the third dorsal and the first lumbar vertebræ, and externally to the investing dura mater, there was a bloody exudation; it was truly an exudation and not a mere congestion. When the dura mater was slit open, a state of very high injection of the minute capillary vessels on the pia mater, which invests the posterior surface of the medulla, extending from opposite the ninth dorsal vertebræ to the extremity of the cord, or cauda equina, was most strikingly apparent to all the medical men who attended the examination. The pia mater which invested the posterior surface of the rest of the spinal marrow was similarly, but in a less degree injected; the injection was most conspicuous at the medulla oblongata. It is to be observed that the appearance now alluded to was limited to the posterior surface of the medulla; it was not seen on the lateral surfaces. On the anterior surface the cellular tissue which lies between the vertebræ and the dura mater, and this membrane itself were very highly injected, (even more so than on the posterior surface) along the whole extent of the spine. When the dura mater was slit open, the arteria spinalis media was found engorged with red blood, and many of its branches, especially at the lumbar region, were in a similar state. The injection which existed on the posterior surface of the medulla was confined to the pia mater: the substance of the medulla itself throughout was altogether natural.

*Cranium.* There was a slight sanguineous transudation between the dura and the pia mater; this latter membrane seemed highly injected at several parts; the thalami optici, corpora striata, and corpora quadrigemina were healthy; but the choroid plexus was much engorged. The pia mater which invests the cerebellum, pons varoli, and medulla oblongata was highly injected over its whole extent; but the substance of these parts themselves appeared to have not been affected.

*Abdomen.* Several portions of the intestinal tube were of an unusually deep red colour; a number of lumbrici were found within.

*Physiological Considerations.* As in the preceding case, the engorgement affected chiefly the pia mater which invested the anterior surface of the tuber annulare, and the posterior surface of the spinal cord, we can at once understand why the spasm assumed the form of general opisthotonos, accompanied with trismus and dysphagia. The anterior surface of the tuber is formed by fibres issuing from the cerebellum; and when these fibres are irritated, they cause spasm of the extensors of the head and neck, trismus, and a spasmodic constriction of the fauces and pharynx. The rigidity of the arms, and the painful difficulty which the patient experienced in attempting to bend them, indicated a spasmodic irritation of the extensor muscles.

The opisthotonos of the trunk proceeded from the congested state of the posterior surface of the dorsal medulla spinalis; and the permanently rigid extension and retro-duction of the lower extremities were owing to the disease having extended to the lumbar portion of the medulla; and the reason of the cramps having been more violent in these than in the upper extremities, is no doubt to be sought for in the more highly congested state of the lumbar, than of the cervical portion. The phenomena of the case are quite in harmony, says Dr. Bellingeri, and indeed corroborate the doctrine, that the cerebellum and the posterior columns of the medulla spinalis preside over the extensor muscles of the body, and do not at all influence the flexors. The absence of any disturbance of the sensibility is to be attributed to the immunity of the substance of the medulla from disease. The history of the case shews that an irritation acting upon the white, or medullary substance, causes spasm, and does not affect the sense of touch; also, that the posterior column of the spinal cord, and the nerves which issue from it, are not to be considered as simply sensory organs, as Bell and Majendie suppose; indeed, the sensibility was little or not at all affected in this child.

Such are the speculations of M. Bellingeri. This is not the place, nor have we at present the disposition, to dispute them. We therefore let them pass for what they are—his opinions.—*Rev.*

*Pathological Considerations.* The vermination is probably to be regarded as the occasional cause which had excited the latent, or masked enteritic affection. Broussais has very justly remarked that diseases of the encephalon and of the spinal marrow are often sympathetically or secondarily induced, by inflammatory or irritative affections of the alimentary tube; the reverse of this position also is equally true. Admitting the correctness of the opinion, that the nervous was consecutive to the enteritic disease, we are led to account for the more marked congestion which was found at the tuber and medulla oblongata, to the more intimate sympathy, and the more immediate connexion which exists between those parts and the abdominal viscera, by means of the great sympathetic nerve. We can also understand how the morbid action may be propagated to the posterior surface of the spinal cord; no doubt in consequence of the numerous communications between this (the sympathetic) nerve, and the posterior fasciculi of the spinal nerves. From these considerations, it must be apparent, how necessary in medical practice it is to have our attention most diligently directed to the state of the primæ viæ, in all affections either of the brain or of the spinal cord.—*Annali Universali di Medicina.*

---

### Cancerous Ulceration of the Nose treated with Creosote.

A young man, 17 years of age, reported that about a year and a half before his application to M. Graefe, a pustule formed on the lower margin of the ala nasi; that he scratched it off with his nail, but that it speedily formed again, and extended, giving rise to an ulceration which eat away all the pinna, and part of the apex of the nose, and attacked also the lower lip; the ulcerated surface discharged an ichorous offensive matter. M. G. when he first saw this patient, discovered, upon looking into his mouth, that on the right side of the palate also there were several small ulcers of a very unhealthy appearance. A variety of remedies had been tried by different surgeons; but the disease had baffled them all. M. G. considered the case as well adapted for a trial of the creosote; he touched the surfaces of the sores in the throat, as well as on the nose, with a pencil dipped in creosote water, and introduced into the right nostril a dossil of lint wet with it. The application caused a slight burning sensation. It was used once daily: on the fourth day, the surface of the ulcer on the lip was observed to be quite dry, and to be covered with a reddish-brown crust. Upon removing this, a few drops of blood flowed from the raw surface. The creosote water was, however, immediately re-applied—the crust was again formed within 24 hours, and again removed. This treatment was continued for four days, and, at that period, the appearance of the sores was decidedly much improved; the surface was no longer fungous, but dry, and, as it were, mummified; the lower margin of the ala nasi exhibited a point of cicatrization, and this sanative process advanced gradually during the ensuing fortnight. M. G. now applied the pure creosote, in place of its solution in water; the pain caused by the application was very smart and pungent; but it did not last long. On the morrow, the crusts were found to be more strongly adherent, and considerable difficulty was experienced in attempting to remove them. The application of the pure oil was continued regularly once a day; and so satisfactory was the progress made under its use, that, in the course of another fortnight, almost the whole extent of the ulcer on the nose and lip was cicatrized. A very small portion remained in a state of suppuration at the date of the report.—*Journal fur die Chirurgie, &c.*

The English reader will scarcely fail to notice, that this was an instance of lupus. Various stimulating and escharotic ointments or washes have sometimes succeeded, and more often failed, in healing this obstinate species of ulceration.

---

### On the Employment of Soot, as a Substitute for Creosote.

M. Bland, physician to the Hospital de Baucaire, was led, by analogical reasoning, to believe that soot might probably have similar effects, as an outward application, to the newly-discovered substance, creosote. The preparation of this (creosote) is not only very expensive, but is extremely uncertain and troublesome; it is obtained by the dry distillation of organic substances. Now it may be observed, that the combustion of fuel (wood), in our grates, is an every-day rude exemplification of the same process; the former, indeed, goes on in closed vessels, and the product is, therefore, more simple and pure; but there can be little doubt that the same constituents are present in the heterogeneous soot of our chimneys. M. Bland communicates the results of his practice in the following reports.

Case 1. A boy, 14 years of age, had been affected, for eight months, with the "herpes squamosus lichenoides" on the chin and lower lip. This dartre presented the appearance of a large grey-coloured crust, hard, and very adherent to the skin, which was stretched, painful, and deeply chapped. The disease had resisted a variety of remedies. He was ordered to use a lotion (made by boiling two large handfuls of soot in a pint of water for half an hour, and then strained) four times a day. Within a fortnight, the cutaneous affection was cured, and the skin had recovered its natural colour and pliancy.

Case 2. A younger brother of the former patient had, for a year and a half, laboured under a most troublesome "tinea favosa," or "favus vulgaris," which had spread over the whole of the hairy scalp. Thick, yellow, honeycomb crusts covered the diseased surface, and a most disgusting smell proceeded from it. He was ordered to have the hair cut close, and to apply bread and water poultices for a few days, in order that the crusts might be detached. When this was effected, the soot wash was to be used frequently in the course of the day. On the fifteenth day after the commencement of this treatment, the disease was quite subdued. The application did not cause any pain.

Case 3. A soldier, 22 years of age, had for two years been affected with an "eruption crouteuse" (impetiginous ?) on the mucous membrane of the nostrils, which had obstinately resisted all the methods of treatment which had been tried. When admitted into the Hospital de Baucaire, on the 21st of last March, the nose was swollen, indurated, and painful on pressure—the upper lip also was swollen—the edges of the nostrils and the mucous membrane, for some extent inwards, were so covered with thick, yellow, honey-comb-like crusts, that the patient could with difficulty breathe through the passage. He was ordered to steam and bathe the parts frequently with the soot lotion, and in the intervals to apply an ointment, composed of an ounce of soot and an ounce of lard. By the fourth day of the following April, the cure was pronounced complete.

Case 4.—*Diptherite.* A young soldier had been affected, for a fortnight, with numerous small and very painful ulcerations on the gums, and on the inside of the cheeks. The gums were soft, ulcerated, and fungous—they were covered with a grey-coloured, fetid, pseudo-membranous layer, which separated in irregular portions; the slightest pressure caused the gums to bleed. This patient was cured in the course of three days, by using as a gargle the decoction of soot.

Case 5.—*Cancerous Ulceration of the Breast.* A woman, 64 years of age, whose catamenia had already ceased for 15 years, applied to Dr. B. under the following circumstances. There was an ulcer, four inches long by three in breadth, and about two in depth, occupying the right mamma; the margins of the sore were hard, knobby, irregular, and everted, of a pale red colour, and exhibiting here and there yellow-coloured points; the discharge was ichorous, and most abominably fetid; the pain which this patient experienced was excessive, and had deprived her of sound sleep for upwards of six months; her health had consequently suffered much, and she was evidently falling into a state of marasmus. She reported that, five years previously, she perceived a hard, moveable tumor, of the size of a walnut, in the substance of the right mamma—that this tumor gradually enlarged, so that, in the course of two years, it was as big as a hen's egg—that, at this period, she began to feel darting pains through it; these were at first slight and intermitting, but they became progressively more and more severe, and at length insufferably agonizing. The nipple was retracted so much as to be scarcely recognizable, and the surface of the mamma was covered with numerous varicose veins, which had occasionally burst, and discharged a quantity of blood. [It is not stated at what period the integuments had given way, and the ulceration had commenced.—Rev.]

The soot lotion was ordered to be applied frequently, and, in the intervals, the sore to be dressed with an ointment, composed of two ounces of lard, two of soot, with two drachms of the extract of belladonna. The patient experienced a most soothing effect from this ointment. In eight days, the size of the ulcer had diminished by two-thirds; the edges had become soft, pliant, and depressed; the fœtor of the discharge was corrected, and the surface was covered with numerous healthy granulations. In the course of another fortnight, the cure was complete.

Case 6.—*Cancer of the Uterus.* A woman, between 60 and 70 years of age, began, two years after the cessation of the catamenia (which took place in her 50th year), to experience a sensation of distressing weight in the genital organs; this was followed by a leucorrhœal discharge, not constant, but recurring at intervals. These symptoms continued unrelieved for several years, without materially affecting the general health; but gradually the sense of weight increased, and it began to be accompanied with sharp lancinating pains within the vagina, and with difficulty in discharging the urine and fæces. When she applied to Dr. B. in December, 1833, the following is the report of her condition. Almost constant darting pains felt within the vagina, from which there was a bloody, fetid discharge—intolerable pain in the left groin (no swelling, nor even redness, was to be perceived there)—shooting to the left hip and thigh ; the effort of relieving the bladder and rectum most painful, violent, and often quite ineffectual, so that the urine required to be drawn off, and emollient enemata to be given, for the purpose of soliciting the alvine evacuations—a sensation of icy coldness in the loins—utter want of sleep—anorexia—great debility, and general decay. Manual examination at once detected the existence of a large ulcer, occupying the cervix uteri, which felt hard, irregular, and tuberculated—the ulcer was nearly three inches in extent, with scirrhous, everted edges. The patient had consulted Professor Lallemand, of Montpellier, who had advised anodyne injections, and the free use of conium, opium, aconite, and other medicines of the same class ; but the relief thus obtained was very small.

Dr. B. ordered the frequent use of the soot lotion, as an injection, on the 11th of March. By the 18th, the characters of the ulcer were decidedly improved, and he then recommended the ointment (as in the last case) to be applied in the intervals. Considerable difficulty was experienced in employing these remedies : with the view, therefore, of assisting their operation, he endeavoured to fumigate the elevated surface with the steam of a strong decoction of soot, introduced by means of a funnel, having a curved pipe. On the 22d of the month, the progress already made was most satisfactory; the edges of the sore had lost much of their hardness and inequality—the tumefaction had considerably subsided, and the fissures or rhagades had disappeared—the pain also was greatly abated; the patient was able to void the urine and fæces with much less difficulty, and she had obtained more quiet sleep than for a long time previously. On the 10th of April, the ulceration was quite healed; the uterus had shrunk so much, that it was not easy to reach it with the finger; but, when it could be felt, it gave the impression of a hard and scirrhous mass. The finger, when withdrawn, was not covered with the ichorous discharge, as hitherto. M. Bland saw his patient a month after this date, and he then satisfied himself, by a most careful examination, that the part of the uterus accessible to the finger had lost much of its induration and diseased feel, and had recovered its normal consistence and pliancy—the cervix uteri had been almost quite destroyed by the ulceration, and even part of the "bas fond" of the womb; of this M. B was made sensible by the remarkable depression, or groove, formed at the lower part of the cicatrix. This cicatrix felt to the finger like a round bag, closed by a piece of cord.

CASE 7—is that of a middle-aged man, who was affected with an obstinate "herpes præputii." The glans and inside of the prepuce were covered with numerous pustules, from which oozed out a fluid, which thickened into soft yellowish crusts ; the itching was intolerable, so as to deprive the patient of sleep. The disease was quite cured, in five days, under the use of the soot lotion.

CASE 8.—*Herpes Squamosus Lichenoides*. A woman, aged 49, had for 12 years been suffering from a dartre, which formed a crust, from four to six lines in thickness, on the upper part of the left cheek, and on the left side of the nose; the surface was deeply chapped, and from the fissures there escaped a sero-purulent matter : the itching was exceedingly distressing, and when the patient rubbed the crust off, the skin underneath was found to be red, granulated, and dotted with numerous yellow points, from which the discharge began to ooze out: the edges were surrounded with an areola of a bright red colour, and the lower eyelid was everted, and dragged so much inwards, as to expose its inner surface, in consequence of the tension of the skin. The crusts were first softened with bread and water poultices, and when they were detached, the use of the soot lotion and ointment was commenced. In between three and four weeks, the dartre on the cheek was almost completely cured, only one long chap remaining—that on the nose, however, was not much changed. In other three weeks, the only trace of the dartre on the cheek was the formation of a thin, yellowish pellicle, not rising above the level of the skin, and adherent to the surface of the cicatrix; the dartre on the nose was diminished in extent, but its surface was still granulated, and of a yellow colour. Ten days after this date, the report states that the cheek was now, to every appearance, quite sound, and that the disease on the nose was advancing most rapidly to a healthy cicatrization.

Case 9.—*Herpes Squamosus Scabioides, of* 40 *Years' Standing.* P. B. aged 60, of an apparently sound constitution, and who had enjoyed exceeding good health on the whole, had for the last 40 years been annoyed with a large, dry, squamous dartre over the whole of the front of both legs, from an inch below the knees to the neighbourhood of the ankles. It was attended with a most troublesome itching, and had resisted most obstinately every plan of treatment. On the 10th of May of the present year, he applied to M. Bland, who ordered him to take a tepid bath or two, for the purpose of softening and separating the scales, and then to rub all the affected parts with the soot ointment. On the 16th, only a few isolated pustules were to be seen over the larger surface which the dartre occupied; in the intervals between these, the skin was of a faint red colour, and was beginning to assume a healthy aspect.

[The progress of this case is not related.]

The other successful cases recorded by M. Bland need not specific allusions, as they are analogous to some already quoted. It may be mentioned that he has derived most pleasing effects from his remedies in pruritus genitalium, and in genuine psora, or scabies. Afraid, no doubt, of the incredulity of his brethren, our author has done well to acknowledge, that he has failed to obtain any curative results in some cases; as, for example, in one of cancerous ulceration of the nose—in one of cancer of the mamma (although, in this case, the soot lotions had the effect of stopping the discharge, and causing the surface of the sore to become dry) —and, lastly, in a chronic ulcer situated on the back of the left hand; the suppuration was, indeed, quickly dried up, but the fore-arm thereupon began to swell, and to become painful and inflamed; these symptoms were at once relieved by omitting the use of the lotion, and by the consequent recurrence of the discharge.—*Révue Médicale.*

The preceding memoir we have given at considerable length, as the author's character stands honorably with the profession. We fear that his success has been too great. How much it is to be regretted that the nomenclature of cutaneous diseases adopted in France is so unmeaning.—Rev.

---

### Ophthalmia treated with Lotions of Corrosive Sublimate.

M. Bally, in a paper published by him a few years ago, in the Gazette de Santé, strongly recommended the employment of a solution of the oxymuriate of mercury in ophthalmia, whether of an acute or of a chronic character. The following two cases are adduced, as confirmatory of its good effects.

A woman was seized, after exposure to alternate heat and cold, with a violent inflammation of the conjunctiva of the left eye. The organ was exceedingly painful, and could not tolerate the impression of light—she felt as if sand had got in between the lids and eyeball, and the tears were hot and burning. The disease had lasted six days, when this patient consulted M. B. who ordered her to bathe the eye, a dozen times at least in the course of the day, with a collyrium, composed of four grains of the sublimate and four ounces of water. In the course of three days, the inflammation had almost entirely disappeared, and by the sixth the woman was pronounced cured.

In the second case, the ophthalmia had continued for eight weeks before the patient consulted M. B. He had been repeatedly leeched, blistered, and also well physicked, but without much benefit. Both eyes were inflamed; and on the cornea of the right one there was a nebulous spot, of the size of a millet-seed;—this eye was more painful than the other, and the sight was more imperfect. M. B. immediately ordered the use of the sublimate collyrium, and a blister on the nape of the neck. No change was made at any time in the treatment; by the 13th day, all inflammation had disappeared from both eyes; the speck however remained; but, fortunately, this also vanished under the application of the "pommade de Janin," and the vision was completely restored. The author adds, that he could cite at least twenty other cases of ophthalmia, treated successfully by the same plan.—*Revue Médicale.*

---

### Use of Chloruret of Lime in Blenorrhagia.

M. Graefe, of Berlin, was among the first to employ this remedy in inflammatory discharges from the urethra; and so favourably did he augur of its good effects, as to state that it would cure the disease when copaiba and cubebs had failed. It was used both internally, either in the form of mixture or of pills, and externally as an injection: the formula for the pills is as follows:—Take of the chloruret one drachm, of extract of opium nine grains, and as much gum as may be necessary to form a consistent mass, which is then to be divided into 54 pills. At first, one may be taken every two or three hours; and the dose is to be gradually increased till eight, ten, or twelve are taken every hour. The injection is made by dissolving 24 grains of the chloruret in six ounces of water, and adding half a drachm of the vinum opii. The strength must be regulated according to the irritability of the canal. This treatment has been successfully adopted in acute as well as in chronic cases; but it is in the latter set chiefly that the greatest benefit has been obtained. As a matter of course, if the irritation produced exceed certain limits, we must omit the use of the chlorurets, and resort to a more soothing treatment. In one patient, who had had a gleet for two years, the discharge was stopped in the course of ten days.—*Trav. de la Soc. Med. de Bourdeaux.*

Our continental brethren discover so many successful remedies, that we wonder how any incurable or obstinate diseases remain. The chloruret of lime may vanquish gonorrhœa in the practice of M. Graefe, but we regret to say, that the English malady refuses to submit to its potent influence. In other words, we have seen it tried for female and for male discharges. It is rather better in the form of an injection, than mere cold water.—Rev.

---

### Observations on the Section of the Tendo-Achillis in Cases of Club Foot.

M. Stromeger, surgeon to the King of Hanover, has the merit of having first experimentally proved, that the division of the tendo-achillis is often by far the most efficacious mode of treating this deformity.

Several cases have occurred to him, in which it has been performed since the date of his last report; and the following contains the substance of a communication from him to the Archives Générales, in the month of June.

H. Leging, a boy seven years old, had been affected with club-foot in both extremities since birth. By the uninterrupted use of appropriate bandages, the deformity had been somewhat corrected, especially in the left limb.—When brought to M. S. the edge of the right foot was found to be strongly turned inwards, the point of the foot directed downwards, and the limb throughout to be considerably emaciated. When he walked, he rested upon the fibular edge of the foot, and his gait was consequently difficult and ungraceful. The voluntary movements of the foot were very imperfect and constrained; but the member was readily brought into a correct position by very gentle traction. The circumstances of the boy's parents being unfavourable to the tedious treatment of such an affection by means of splints, &c. applied for a length of time, M. Stromeger recommended that the tendo-achillis should be divided, for the purpose of equalizing the action of the flexor and of the extensor muscles, between which two sets of muscles the normal antagonism had never duly existed.

The operation was performed on the 26th August, 1832; in five days the outward wound had cicatrized, but the extension of the limb was deferred for other three days, as the cicatrix remained somewhat tender. The foot was then easily brought into the proper position, and there retained, by "une extension croisante," for three weeks—the angle which it formed with the leg was about 70°. The apparatus being then removed, M. S. found that the substance intervening between the cut ends of the tendon was shorter than he had expected; it was not more than a quarter of an inch long, and was much thinner than the tendon itself. The foot, for a few hours, was retained in a correct position; but then it was gradually drawn into the former faulty direction, notwithstanding the use of a shoe, provided with a steel rod on the outer side. The condition of the boy was therefore not at all mended; and M. S. was anxious to repeat the operation; but to this the parents of the child would not accede.

The failure in this case is to be attributed to the delay of commencing the extension of the limb. By the eighth day, the adhesion between the two cut ends of the tendon had acquired too much strength.

*Case* 2. H. Linse, 13 years of age, has laboured under club-foot, on the left side, for nine years. No satisfactory cause could be assigned for its occurrence. No treatment had been followed in this case, and the condition of the boy was gradually becoming worse. In August, 1833, he was taken to M. Stromeger for advice. At this time, the act of walking was so difficult and painful, in consequence of the tender state of the outer edge of the foot, on which all the pressure was made, that he seldom ventured out. The toes, and more especially the big one, were drawn strongly inwards, the tendon of the flexor longus pollicis being in a state of constant and powerful tension. It required, therefore, considerable force to bring the foot into a straight direction.

M. S. deemed it expedient to divide the tendon of the flexor pollicis in the first place;—this he did by introducing the point of a knife behind the tendon on the inner edge of the foot, midway between the heel and the great toe. The limb was allowed to remain quiet for three days; and it was then fixed in an apparatus fitted to keep up a constant extension. In the course of a week, there was a very decided improvement in the direction of the point of the foot; and now the section of the tendo-achillis was performed. A splint and bandages were applied. The outer wound was healed by the fifth day, and extension was then commenced. In ten days, the foot formed with the leg an angle of 70°. The apparatus was not removed till the end of the fourth week after the operation; and then the boot, which Dr. S. recommends, was substituted, and a continued extension ordered to be kept up during the night. The young patient began to walk with great ease, and was permitted to go out for a short time on the third day after the removal of the apparatus. The cure was progressive, and eventually complete.

*Case* 3. Ferdinand West, nine years of age, had club-foot of the right side at birth. By constant attention, the condition of the little patient had been prevented from becoming worse. When five years old he was taken to Dr. Stromeger, who recommended the use of a peculiar apparatus, by means of which the foot was brought into and retained in a straight position. The use of this was continued for five months; and after that time, Dr. S. lost sight of the boy for the next four years. When again brought to him for advice, he found that the state of the foot was nearly the same as when he had last seen him, and the walking was even more difficult, in consequence of the pain which it occasioned. The dorsum pedis formed quite a convex arch, the toes being pointed down, and drawn in towards the heel, with the exception of the big toe, which was drawn upwards. The division of the tendo-achillis was performed on the 10th of January; the wound being healed in five days, the extension was commenced, and continued uninterruptedly for four weeks; the boot was then substituted, and the child permitted to walk. The facility with which he did this was most satisfactory. As the point of the foot was, however, still drawn inwards by the action of the flexor longus pollicis, Dr. S. divided the tendon of this muscle, and three days afterwards the tendon of the extensor pollicis: the foot was replaced in the apparatus, and the great toe forcibly drawn forwards. In a week after this operation, the patient was allowed to use the boot. The section of the tendons of the chief muscles of the great toe, so far from paralysing its movements, had caused them to be more easy and free of execution; and the limb gradually acquired greater strength than it had before.

*Case* 4. Mademoiselle B., 19 years of age, had laboured under scrofulous disease in her early years, and had at one time lost the use of both her limbs. This paralysis had gradually ceased, but had left behind great weakness of the right extremity, the foot of which became contracted. The expedients which had been resorted to for the cure of this deformity had failed. When she consulted Dr. S. he found that the dorsum pedis was much arched downwards, so as almost to obliterate the hollow of the instep;—the inversion of the foot was not considerable. The point d'appui in walking was the outer edge of the metatarsal bone of the little toe. The heel of the boot which she wore required to be between four and five inches high. It was not easy to bring the foot into a correct position, even when considerable force was used. The tendo-achillis was divided on the 11th of March; on the 16th the continued extension was commenced.—Three weeks after the operation, the foot formed with the leg an angle of 70°. The apparatus was then exchanged for a boot, provided with a steel rod on the inside; and the young lady began to take gentle exercise. The act of walking was at first extremely difficult and painful, but by degrees became more easy and perfect; and at the date of the report, she had gained almost completely the healthy use of the limb.—*Archives Generales.*

We give this memoir as we find it. We do not pledge ourselves to the opinions expressed by the authors in these foreign articles. Absence of criticism must not be considered as actual assent.

---

Case of Spontaneous Dry Gangrene, cured by Bleeding.

A countryman, 40 years of age, admitted into the Hotel Dieu, presented in the hands and feet that rare variety of gangrene which some authors have denominated "white gangrene." The fingers were pale and of an icy coldness; the second and third phalanges were wasted, and, as it were, mummified; the form of each finger was conical or fusiform; the nails were puffed out and had a chalky appearance; the skin round their bases was slightly ulcerated. The last phalanges were completely insensible; in the others, the patient felt a sense of formication and extreme cold; he had no power of moving the fingers; the skin was white and dry like parchment, and emitted no gangrenous smell. The appearance of the feet was nearly similar to that of the fingers; presenting the same icy coldness, the loss of all voluntary motion, and the absence of pain. The ears, nose, and lips of this man exhibited a remarkable deficiency of their usual warmth and colour. The action of the heart was vigorous, but rather irregular. The other functions of the body were in a normal condition; the patient said that he felt very well inwardly, and that his only reason for coming to Paris for advice was, that he had been unable to use his hands at any work for the preceding four months. The previous history of the case, as far as it could be well gathered from the patient's account of himself, was as follows.

The disease had commenced four years ago, but had varied a good deal in intensity at different times, according to the season of the year; alternately abating and increasing. It was generally worse during the heats of Summer, especially when the patient was exposed to the direct action of the sun. During the first years, this species of glacial torpor, or asphyxia, did not last beyond a few days; the fingers however did not regain at any time their natural warmth and flexibility.

No satisfactory cause could be assigned for this singular disease. Rigid enquiries were made respecting his food, but nothing satisfactory could be ascertained to account for it. His occupation had been that of a thresher of corn. His general health had been, as already stated, perfectly good.

M. Dupuytren, regarding the disease as an inflammatory affection of the arterial capillaries, akin in its nature to what takes place in the gangrena senilis, resorted to the same mode of treatment, which he has been in the habit for many years past of employing so successfully in the latter species. The patient was bled from the arm, poultices were applied to the parts, and a moderate diet enjoined. In the course of eight days, the heat, colour, motility, and sensibility of the hands and feet were restored, as if "par enchantement." The patient was advised to have recourse to the same treatment, if the disease should ever return—*Revue Med.*

### Hydro-bronchocele, or Hydrocele of the Neck.

A man, 50 years of age, entered the Hôtel Dieu, for the purpose of having a tumor, which impeded his breathing, removed from the front of the neck. It was of the size of an orange, situated between the os hyoides and the thyroid cartilage; the superjacent skin was healthy; a distinct fluctuation might be perceived. A finger, passed deep into the mouth, could feel the base of the tumor, behind the tongue. The breathing was sometimes so obstructed, that he was in constant dread of being suffocated; and his speech was so affected, that he could only with great difficulty pronounce any long word.—M. Dupuytren plunged a bistoury into the most projecting point of the tumor, and discharged a large quantity of a thick, yellowish-coloured fluid; the incision was enlarged to the extent of an inch and a half, and when the cyst was well emptied of its contents, some lint spread with simple ointment was introduced into its cavity, with the view of inducing a certain degree of inflammation, in the hopes of obliterating it altogether. The progress of this case was quite satisfactory, and the patient was relieved of an annoyance which had afflicted him for a great number of years, and which of late had seriously affected his health.

*Reflections.* Hydrocele of the neck, of which the preceding case furnishes a good example, has often been stupidly confounded with some varieties of genuine bronchocele. Foderé has fallen into this mistake in his treatise on the goitre. It is quite true indeed that the thyroid gland becomes sometimes degenerated into a morbid structure, made up partly of cysts, which are filled with fluid; but such cases are quite distinct from the disease, to which the term "hydrocele of the neck" has been, and ought to be, applied.

The latter disease is a simple encysted watery tumor, and may very generally be cured in the manner above described; whereas the encysted bronchocele is a much more intractable disease, in consequence, no doubt, of the other morbid structures which are co-existent.

The only danger attending the incision of, and attempts to obliterate encysted tumors of the neck, arises from the hazard of the inflammation excited in the sac being propagated to the air-passages.

The author who first accurately discriminated and described this disease is M. Maunoir, of Geneva. He pointed out the analogy between hydrocele of the tunica vaginalis of the testicle, and the watery encysted tumors in the neck. He tried the effects of injecting stimulating fluids into the sac, after the withdrawal of its contents; at first he reported favourably of his trials, but he has since changed his opinion, and he now positively denounces the practice as generally inefficient, and sometimes highly dangerous; in one case, dreadful pains, convulsions, trismus, and diffused abscesses were the results of the experiment, and the desired object was not attained; for the disease returned after these alarming symptoms had been subdued. He does not approve of Dupuytren's method, but recommends that, in all cases, the seton is to be preferred, assuring us that its use has been eminently successful in his practice. Six or eight weeks are generally sufficient for the cure.—*Révue Médicale.*

---

### PARALYSIS INDUCED BY THE PREPARATIONS OF LEAD.

In every one of 17 cases of this disease, observed by M. Tanquerel, the paralysis affected the upper extremities—in five only were the lower limbs affected at the same time; in five, aphonia, or some impediment of speech was present; and, in one, the intercostal muscles, the pectoralis major, and sterno-mastoideus appeared to have lost their contractility. Although, generally, the paralysis is limited to the muscular system, without disturbing the sensibility of the parts, yet cases sometimes occur, in which this latter function is injured simultaneously; it may be either exalted, impaired, or altogether abolished. The first of these morbid conditions is the most common.

The muscles most frequently affected are the extensors of the fingers, the supinators, and the adductors and abductors of the thumbs. When the lower limbs are the seat of the paralysis, there is almost always some affection of the upper ones at the same time. As stated above, the tongue may become completely or partially paralyzed, and the patient will then stammer, or even lose all power of articulate speech; or the muscles of the larynx may be affected, and the voice be quite lost. The paralytic symptoms induced by lead generally come on very gradually, and almost imperceptibly, as the colicky symptoms abate and disappear; at other times, the colic and paralysis are simultaneous in their attack, and the former may be relieved, while the latter seems to be not at all affected; and, lastly, the paralysis has been known to supervene suddenly, during the severity of a colic attack, banishing it, and assuming, as it were, its place in the system; but this is a rare occurrence. One practically important fact has been often overlooked by authors, namely, that the saturnine paralysis may be induced, without having been preceded, or without being accompanied, by any affection of the intestines. Its duration varies from a few days to several years. As a general remark, it is worthy of notice, that most cases of lead palsy are, at least in their early stages, susceptible of cure.

The remedies which have been most approved by experienced men, are electricity, sulphureous baths, and the internal and external use of the nux vomica. M. Rayer, the distinguished physician of the La Charité Hospital, has been eminently successful in the treatment of this disease; he employs the strychnine, first internally, and then in the endermic method, conjoining the daily use of sulphur baths, or fumigations. The pathology of lead palsy is not at all known. "Would to God" (exclaims a French reviewer of the Hippocratic school) that many other diseases, so well known in an anatomical point of view, were as easily cured." —*Essai sur la Paralysie de Plomb. Paris*, 1834.

---

### Literary Curiosities.

An ingenious contributor to our valued contemporary, the Revue Medicale, has endeavoured to lay down and illustrate certain rules or maxims to be followed by those who are much engaged in mental occupations. Among other topics which he has introduced, he alludes to the very different habits of different authors, when occupied in composition. Montaigne shut himself up in an old tower "pour y digerer librement à loisir ses pensées." Rousseau herborized; it was, he used to say, "en se meublant la tete de foin," that he could think most profoundly. Montesquieu composed the groundwork of the "Esprit des Lois," while reclining in a post-chaise. Milton generally composed at night, sitting in his arm-chair, with his head resting on the back of it. Bossuet sat in a cold room, but kept his head warm with a quantity of coverings. Mr. Fox, after having indulged to excess in the pleasures of the table, would often, when he went home, retire to his study, and wrapping a cloth dipped in vinegar and cold water round his temples, sit engaged in study for ten hours successively. On the other hand, we are told that Schiller wrote most of his best pieces when he had his feet immersed in ice-cold water. Maturin, the author of Bertram, Melmoth, &c. withdrew into the most retired privacy when engaged in composition; when the inspiration seized him he used to place a wafer between his eyebrows, to announce to his servants that they were not to disturb him. Jeremy Bentham was in the habit of writing all his ideas on small scraps of paper, and then stringing these together, so that they resembled rather a huge file of a merchant's bills, than the manuscript of an author's work. Napoleon, we are told by Bourrienne, when engaged in deep thought, would often be humming or singing a tune all the time, or notching the arm of his chair with the "air d'un grand enfant;" then suddenly he would start up, and point out the design of a monument to be built, or explain some of those mighty projects which used to astonish and terror-strike the world. These from out an almost infinite number of examples, sufficiently prove how different in different men are the circumstances favourable to mental labour; no general rule can be laid down, and the only advice to be suggested is, that each must work according to his fancy, habits, and ease.

What has been said of literary composers holds equally true of their musical brethren. Some, as Sarti, &c. can compose only in silence and gloom. Cimarosa delighted in noise and brilliancy. Sacchini found relief, and almost assistance to his ideas, if several kittens were playing in the room beside him; and Paesiello, in his fits of composition, used to bury himself under the bed-clothes, trying to banish from his memory all the rules and precepts of his art, and giving vent to his feelings in the exclamation "Holy Mother, grant me the grace to make me forget that I am a musician." !!

It is an observation as old as the hills, that great and enduring works can be achieved only by patience and tedious thought. Perfection in any accomplishment, said Girodet, "ne s'improvise jamais." And Antoine de la Salle used to remark of any one commencing a work of importance, "celui n'est que l'ecolier de celui qui le finit." Even Voltaire, who perhaps of all authors had the greatest facility of rapid composition, was well aware of the requisite labour which must be spent in frequently revising his work; writing to M. Dargental on the occasion of a tragedy which he had very quickly composed, he wittily says, "Ma tragedie est finie; mais vous sentez bien qu'elle n'est pas faite. Mon ours de six jours demande six mois à etre leché."

But the tedious labour of long-continued and of often-repeated thought, is by no means the attribute of the authors of the present times; men of science, as well as men of literature, are in a mighty haste after fame and glory, and there is many an "esprit genereux," who, like Champollion, is feeding himself with the hope of leaving "sa carte de visite chez la posterité." Our ingenious cotemporary concludes his amusing paper with some sensible remarks on what may be termed the "adjuvantia" of mental labour; having alluded to the advantages of an airy, cool, and quiet study, he then, en passant, suggests the propriety of wearing large, wide, and convenient articles of dress (every one knows "les Regrets à ma vieille Robe de Chambre," one of Diderot's best pieces), and points out the injurious effects of stooping too much, or for a great length of time, at a low table, when engaged in writing. It is well (says he) to rise from the table frequently to walk about the room, read aloud, and occasionally to vary the subject of meditation by one of a less grave character. These precepts, although seemingly trifling, are not so in reality. "An atom makes a shadow," was an observation of Pythagoras; and there is as much physiological as mere physical truth in the observation; the slightest organic lesion, the smallest injury, the least perceptible disturbance of health, may become, if neglected, the parent of much serious mischief.—*Revue Médicale.*

---

CHEMICAL EXAMINATIONS OF SPOTS, CAUSED BY BLOOD AND SEMEN, ON LINEN.

A charge of attempted rape on the body of a young girl, 11 years of age, was lately preferred against a man at Paris, and M. Chevalier was applied to, for the purpose of ascertaining, if possible, the nature of certain spots which had been found on the chemise of this child. The following is an account of his researches.

1. *Spots caused by a Bloody Fluid.* The linen so stained was cautiously immersed under the surface of distilled water; in the course of a few minutes, a brownish cloud began to separate from the linen, and was gradually precipitated to the bottom of the vessel, in long reddish streaks. By the end of an hour, the water had acquired an uniformly reddish-brown colour—it restored the colour of paper which had been reddened by an acid; but this property seemed to be quite independent of the admixture of any fluid, and was no doubt caused by the alkaline impregnation which all linen receives when dressed. Heated to 100° C. in a glass tube, the discoloured water became more cloudy, and yielded a reddish-grey coagulum, which, on the addition of a few drops of caustic alkali, was re-dissolved, leaving the fluid of a greenish hue by reflected light, and of a reddish-brown, by refracted. An infusion of gall-nuts caused a reddish-grey precipitate.

2. *Spots produced by the fæculent Discharges.* The linen was treated with water, in the same manner as in the preceding case. The water speedily acquired a yellowish colour, and the characteristic smell. The alkaline property was again noticed, and arose, no doubt, from the cause which we adverted to. The filtered solution gave a precipitate with infusion of galls; evaporated in a glass dish, an excrementitial odour was emitted, and an albuminous coagulum furnished; this was mixed with a greenish-yellow matter, slightly acid and "sucrée," analogous in taste to picromel.

3. *Spots produced by an Animal Mucus.* Linen stained with seminal fluid, when immersed under the surface of water, did not, as in the preceding cases, induce any disturbance of its transparency; it (the linen) became slippery and viscid when moistened; the water yielded a few flocculi on the addition of nitric acid—when evaporated, it gave no coagulum, and emitted a peculiar faint smell, somewhat like that of fresh semen. Now the stains on the linen of the girl gave rise to appearances which did not agree with those now mentioned; the water with which it was treated became troubled and cloudy, and a flocculent precipitate was formed at the bottom of the vessel; the linen, when moistened, did not become viscid to the finger; the precipitate caused by the addition of nitric acid was much more copious than had been the case with the sperm-stained linen, and the liquid, when evaporated, became coagulated, and gave out an odour of animal gelatine. These negative proofs were made more satisfactory, by comparing the results with those obtained from linen stained with a leucorrhœal discharge; the same precipitation by nitric acid, the coagulation by heat, and the giving out of an odour of animal gelatine.

The conclusions which M. Chevalier arrived at were—

1. That the red stains on the chemise of the girl had been caused by blood.
2. That the alkalinity of the water in which the linen had been immersed was of accidental occurrence, as other linen, free from all stains, produced the same phenomena.
3. That the yellow spots on the chemise had been caused by the fæcal discharge.
4. That the other stains had been caused by the vaginal mucus, and not by seminal fluid.—*Journal de Chimie.*

---

Analytic Epitome of the Second and Third Sections of M. J. B. Cayol's Traité des Maladies Cancereuses. Paris 1833.

Cayol, in the second and third grand divisions of his Treatise on Cancerous Diseases, a materially improved edition of which is now before us, passes in review the therapœia and empirical nostrums, whether external or internal, which have obtained notoriety in France, Germany, and Great Britain.

In this article, it is intended to notice the most important of M. J. B. Cayol's remarks, particularly in reference to his unwearied research, and success in imparting a knowledge of the ingredients of the recipes of Dubois, Bayle, Plenck, Storck, Lambergen, Gerbier, and Gamet, &c. With the only other prefatory remark which we deem it necessary to make, that a leading object with us is brevity, we proceed at once to the—

First Chapter.

*External Remedies.*

*Arsenical Preparations.* Of these, the white arsenic of commerce is unquestionably the most efficacious. It does not cure the disease, but acts on the degenerated parts, which it destroys by its energy, as an escharotic. It was first used by Fusch, in 1594. His formula was composed of white arsenic, chimney soot, and great serpentary root. Its application to ulcered surfaces was occasionally followed by fever, accompanied with rigors, vomitings, fainting fits, requiring the renouncement of the remedy.

*Rousselot's and Frère Come's Powders.* The merit of these powders consisted in different substances being combined with the arsenious acid, by which its deleterious effects on the system were obviated, without impairing its action as an escharotic. Rousselot's powder was composed of *dragon's blood and cinnabar, of each two ounces—white arsenic, two grains. The whole mixed, and thoroughly pulverized.*

Frère Come made use of similar ingredients in different proportions, adding to the melange grs. xviij. of the powder of " Savatte brulée."

Professor Dubois for many years made use of this formula—

*Dragon's blood,* ℥j.
*Cinnabar,* ℥ss.
*Arsenious Acid,* ʒss.

*Reduce the whole to powder, and mix thoroughly.*

When about to use the above, Dubois added saliva, in sufficient quantity to form a paste, with which the crusts and excrescences on the surface of the ulcer were smeared; and above this he uniformly applied a stratum of arsenical paste, about two lines in thickness, which he covered with cobwebs (toile d'araignée.) The eschar separated about the twelfth or fourteenth day. The same remedy was advantageously employed upon the wound resulting from an extirpated *cancerous* tumor, when the disease threatened to repullulate.

In the middle of the seventeenth century, the famous powder of Pierre Alliot made a great noise: from the testimony of — Vacher and others, it was a preparation of arsenic, no way superior to the arsenical paste. The same may be said of the solution of arsenic, used in England by William Shearly as an escharotic.

*Preparations of Lead.* To discuss scirrhous *engorgements,* and even cure ulcered cancers, saturnine extract, and other preparations of lead, were much praised by Goulard, physician in Montpellier, and many English and German practitioners.

Marvellous properties were attributed by Brambilla to a plaster, composed of red oxide of lead, olive oil, and turnip-juice. Lead, however, in Cayol's opinion, can be regarded only as an admirable sedative; for, says he, "the observations of these physicians prove that they gave the name, *scirrhus,* to engorgements not of a cancerous nature."

A preparation frequently employed by Bayle with advantage was this—

*Golden litharge,*

*Vinegar, of each,* ʒvj.

*Good Olive oil,* ℥ij.

The litharge triturated in a porcelain vessel, adding the vinegar gradually; next the oil, drop by drop, "continuing the trituration until the mixture has acquired the consistence of half-congealed oil. He spread this liniment over the whole surface of the ulcer by means of a pencil, or with the brush of a quill. By the addition of a sufficient quantity of virgin wax, it may be converted into an ointment. The remedy is particularly advantageous to lull the pains of cutaneous cancer." Vegeto-mineral water is likewise employed with the same view.

*Preparations of Iron.* Mr. R. Carmichael, of Dublin, in 1806, published an essay *On the Properties of Carbonate of Iron,* applied to the treatment of cancerous maladies. Five cancerous ulcers of the face and of other parts, he assures us, were completely cured by being sprinkled with this salt, very finely powdered. Dr. Hall, of London, doubted the nature of the maladies cured by Mr. Carmichael; but he regards carbonate of iron as a valuable remedy in the treatment of phagedenic ulcers, simulating cancer.

*Mercurial Preparations.* The solution of hyperoxygenated muriate of mercury, recommended by A. Wilson, vapour of cinnabar, &c. which have been vaunted as *anti-cancerous,* have never cured *cancerous* diseases; but *venereal* affections only, which, by degeneration, have assumed the appearances of cancers. Cayol makes an important remark—"Mercury, in whatever form administered, has always appeared to us to be hurtful, in diseases truly cancerous."

*Alkaline and Acid Substances.* These have been employed in turn, in accordance with the ideas adopted, as to the nature of cancer. M. Martinet, rector of Soulaines, pretended to having cured many occult cancers, by compresses steeped in a solution of ammonia; drops of the alkali were prescribed at the same time internally. Solution of potass was prescribed by Dr. Barker.

Carbonic acid gas was confidently proposed by Peyrilhe (who never could see any thing in cancer but an *alkaline acrid),* not only as palliative, but as calculated to effect a radical cure. Oxygenated muriatic acid was spoken of in high terms about the same epoch; but of the much that is written, little is trustworthy.

*Animal and Vegetable Substances.* With regard to these, as in the case of alkalis and acids, their ephemeral reputation is partly owing to hypothetic fancies, charlatanism, and fortuitous circumstances.

*Sedum acre,* first recommended by Quesnay, has very recently been noised abroad by Lombard, chief surgeon to the Military Hospital of Strasbourg, who recounts many remarkable cures effected by this plant, fresh and applied to ulcers regarded as cancerous. Solander and Colden had formulæ, in which the juice or extract of the *phytolaca decandra* was the chief ingredient. Expressed juice of foxglove (fresh) diluted in the proportion of a spoonful to a pint of water, to be used in soaking compresses. Gastric juice of animals, (Senebier of Geneva.) Ox blood proposed by Vanevy as a succedaneum for gastric juice.

Cataplasms of the pulp of carrot were eulogized A.D. 1766, as very efficacious, especially in cancer of the breast, by Sultzer, chief physician to the Duke of Saxe-Gotha. This remedy was abandoned, from want of success, in the practice of the French; but, in the interim, Bridault submitted it to experience, with the precautions adopted by every judicious practitioner: and in 1802 he published, what an experience of five and thirty years had apprized him, as to the medicinal properties of carrot.* Cayol, from many trials of this root, coincides with the observations of Bridault, viz. that "carrot cataplasm is inefficacious against cancer; but that it can ameliorate, and even cure, many dartrous, scrofulous, or other affections, which sometimes possess all the appearances of cancer, and which frequently determine this unfortunate organic degeneration in predisposed subjects."—*Clinique*, *p.*521.

Opium, hemlock, hyosciamus, and belladonna, have likewise been used in the form of cataplasms, plasters, fomentations, &c. &c.

M. Steidèle indulged sanguine hopes of his ability to cure cancer, whether occult or ulcered, by the persevered-in application of compresses steeped in liquid laudanum, provided, (added M. Steidèle, and what an important *qualifier!*) "that the patients were not old; *and*, that their *viscera were in a sound state*." Cayol quotes, *Journal de Med. tom.* 82.

*Cauterization.*—Confessedly the first mean resorted to for destroying cancer, was the *actual cautery, (Hippoc. Epidem. Lib.* vij.) M. Leconte hit upon the ingenious idea of cauterizing the lower lip by solar heat, placing the affected part under a very powerful lens;—the operation was very successful.† This practice, more curious than useful perhaps, is thus commented on by Cayol:—"It is certain that this kind of cautery has not, like the actual cautery, the inconvenience of losing a part of its heat, before acting to the depth required; nor that of burning the neighbouring parts; but a solitary observation does not suffice for appreciating an operative process; and it does not appear that this possesses any advantage over the arsenical paste."

*Electricity.*

Dr. Easton (Dublin) relates the case of a lady, who, after having been capsized by a thunderbolt, saw, to her infinite surprise, a scirrhous tumor of the breast disappear. No remedy had been employed.

Cayol judiciously withholds his assent from the conclusion, arrived at by other authors, that electricity should be numbered among the curative means of cancer. "What comparison could be established between the effects of electricity with the precautions necessary not to compromise the life of patients, and the commotion produced by a clap of thunder?"—*Clinique, &c. p.*523.

*Different Topical Applications.*

The reputation of *antiseptics* or *detersives* has been founded on erroneous diagnosis.

Standard authors having asserted that cancer in the last stage has been cured by Plenck's vulnerary water, it would be inexcusable not to indicate its composition. Take of—

*Lime water, one pound,*
*Burning soot from an oven, an ounce,*
*White lead, half an ounce,*

Mix and boil for quarter of an hour, and add—

* Traité sur lar carotte, 8vo.

† Mémoires de la Societe Royale de Médécine. An. 1776.

*Liquid myrrh, half an ounce.*

Compresses or charpie steeped in this liquid were applied to the surface of the ulcer.

Cayol closes his section on external remedies after observing, that of the innumerable list of topical applications, most of them act as escharotics or caustics; "and of the whole of these we know none preferable to arsenical paste;"—others, for the most part, are purely empirical nostrums. It would be impossible to enumerate every pharmaceutic preparation employed as a calmative: our author contents himself with naming two of the most celebrated—the plaster and liniment of Pissier.

*Plaster.* Take of—

*Oil of linseed, two pounds,*
*Red lead,*
*White lead,*
*Yellow wax, of each eight ounces.*

Make, *secundem artem,* into an ointment.

This is spread over the skin, "and is applied to occult cancer, with the view of tranquillizing pain, and preventing ulceration."

*Liniment.* Melt together—

*White wax, two ounces.*
*Linseed oil, four ounces.*

When cool, add *tincture of opium an ounce.*

Spread upon lint, and use as dressings for ulcered cancers.

SECOND CHAPTER.

*Internal Remedies.*

It would appear that the ancients did not employ internal remedies in treating cancerous diseases. Tumors of the mammæ, accompanying a kind of false pregnancy, were attempted to be discussed by reinducing the catamenial excretion. Hippocrates directed a decoction of elaterium to be quaffed, if the cancer followed a diarrhœa or rebellious cough; and, that the ulcer should be dressed with the dust of calcined copper.

Celsus says expressly that cancers which neither fire nor iron can heal are incurable.

If cancerous tumors attained a certain volume, Galen admitted their nature to be incurable, and contented himself with indicating such remedies as were calculated to check their progress.

In modern times a multitude of substances have successively been extravagantly praised in the internal treatment of cancer. Not one of them can be regarded as a specific. The majority of external remedies in different doses and forms have been administered internally. Storck's preparation of the *extract of hemlock* is unquestionably the most celebrated of these.

*Hemlock.* Cayol describes minutely the necessary pharmaceutic processes from the time proper for gathering this valuable plant till ready for being prescribed. The hemlock (conium maculatum) flowers in May and June, when it should be gathered and pounded with a wooden pestle in a marble mortar—next submitted to pressure—the juice passed through a strainer—thickened at a slow fire, to the consistence of thick extract; and, lastly, mixed with a sufficient quantity of the dried leaves reduced to powder, to give it a pillular consistence.

One or two grains of this extract are commenced with evening and morning, gradually increasing the dose to a drachm and a half or two drachms daily. After each dose, the patient should drink a cup of tea, veal broth, or elder-flower water. In general, to obtain good effects from hemlock, the dose should be raised until slight vertigo, trembling, disagreeable feelings in the eyes, or diarrhœa, result. Should these symptoms be carried the length of poisoning, acids, emetics, &c. must be resorted to.

Should the cancer be external, various preparations of hemlock may be used locally; the leaves being applied in their natural state, or as cataplasms. The decoction serves for fomentations or injections. When the dried leaves only can be had, a small bag may be filled, dipped in boiling water, and applied to the part affected, when at a tepid temperature.

Hemlock plaster may be applied on indolent or somewhat painful scirrhi.

Such are the preparations with which Storck (believed at least that he) cured cancers of the breast, womb, &c. towards the middle of last century.—Cayol justly remarks—"A circumstance which should encourage suspicion of the truth of Stork's observations, is, his having cited cases of cure only, thus insinuating, against all probability, that no cancerous disease had withstood his remedy," (p. 529.) The great name of this physician gave an extraordinary popularity to hemlock, and the *anti-cancerous* properties of this plant were supported by multiplied experiments made in every country.

No good ensued, on the assertion of Dehaen, from its use in one hundred and twenty cases. Eight cases of uterine cancer treated in accordance with Storck's method were not ameliorated. Fothergill in England, Bierken in Sweden, Akenside, Rikmann, &c. &c. regarded the extract as ineffective in the treatment of cancer. In the hands of others, notable relief, but never a cure, followed its employment. "Some went even so far as to say that it exasperated the evil at the very time while seeming momentarily to mitigate it." Cullen and many French practitioners agreed as to the "virtue" of hemlock, in curing certain scrofulous and syphilitic "engorgemens." Of later years, M. Alibert repeated with care Storck's experiments upon more than a hundred women affected with ulcers of the womb, &c. "without deriving the least advantage."—*(Nouv. Elem. de Therap. tome* i. *p.* 425.*)*

From these and other observations, Cayol deduces that "extract of hemlock acts, in the first instance, as a stimulant, and afterwards as a narcotic, when the dose is raised to a certain point. It appears of use in facilitating the resolution of many kinds of chronic *engorgemens,* particularly scrofulous and syphilitic affections. It never cures scirrhus nor cancer; but occasionally checks their progress, and renders them less painful. In the latter cases it appears to us to act by modifying advantageously the chronic inflammation of different tissues surrounding the degenerated parts. If, after producing this happy effect, the remedy continue acting as an excitant, it almost always irritates the cancer, and accelerates its progress." (P. 530.)

*Belladonna, Hyosciamus, &c.* Lambergen prescribed a scruple of the dried leaves of belladonna in ten cupsful of boiling water. The patient commencing with a cupful of this infusion every morning fasting; the dose gradually augmented, till dryness of the throat, nervous symptoms, &c. required the suspension of the remedy. This plan was modified by Darluc, Amoreux, Marteau de Grandvilliers, Campardon, and Cullen. It would appear that, when circumspectly employed, belladonna, like hemlock, accelerated the cure of many kinds of lymphatic *engorgemens* simulating cancer, and checked the progress of some diseases of a truly cancerous nature.

The same may be said of hyosciamus, wolfsbane, water-fennel, cherry-laurel —"very active medicines, and the employment of which requires so much the more prudence, as their physiologic and therapeutic effects have not yet been suitably appreciated"—(p. 531.)

### Salts and other Mineral Substances.

*Acetate of Copper,*

Or *verdigrise,* was the chief ingredient of Gamet's remedy, and of the pills of Gerbier—two recipes which in turn enjoyed great celebrity in curing scirrhi, cancers, and inveterate ulcers of the breast and womb. M. Mitag-Midi, a celebrated physician, who had strong prejudices against preparations of copper, assures us, nevertheless, of having seen many cancers of the womb, throat, axillæ, groins, and mammæ, cured by Gamet's remedy.

Dr. Solier of Romillais, by order of the faculty of Paris, made numerous experiments with the preparations of copper, in order to estimate their value as anti-cancerous remedies. An admirable report was published in 1778 & 9 at Paris. It resulted from these experiments, that verdigris produced no effect on cancer of the breast; but acted more beneficially, perhaps, than any other remedy on *cutaneous* cancers. When the dose required being pushed beyond ten or twelve grains, precordial anxiety, colic, obstinate diarrhœa, vomitings, &c. resulted. It has been suggested to renew experiments with Gamet's and Gerbier's recipes thus modified.

"Take of acetate of copper and iron filings, of each two scruples and a half. Triturate these substances for a sufficient time in a copper mortar with a pestle of the same metal. Add one drachm of ext. of hemlock, mix thoroughly, and divide into pills of a grain each." Begin with one of these daily, augmenting the dose cautiously up to twelve or even fifteen grains. Acetate of copper during the whole may, as by Gamet, be applied topically in the form of ointment or injection.

*White Arsenic*

Was announced in 1775 by M. Lefebvre de Saint Ildefond as an *experienced remedy* in curing occult or ulcered cancer.

Four grains of it were dissolved in a pint of distilled water, of which the patient took a spoonful every morning, united with an equal quantity of milk, and half a drachm of syrup of diacordium. Eight days thereafter (there being no contraindication) a second dose was given in the evening, and perhaps a third at mid-day. After the contents of the first bottle had been consumed, a second quantity was prepared from six grains instead of four; and eight grains was the maximum. Six bottles sufficed in general in curing cancer of the breast. The ulcer was bathed daily with a solution of arsenic (viii. grs. to the pint) and covered with a cataplasm of carrot pulp, boiled in the same solution, to which were added sugar of lead, laudanum, and extract of hemlock, in varying proportions.

In 1778, M. Roennow published the results of his experience. During fifty years trial, says Cayol, "of this remedy, he averred having cured thirty well characterised cancers; in short, he did not hesitate to announce arsenic as a specific against the cancerous vice." After alluding to such positive assertions in connexion with "les annonces fastueuses des charlatans," Cayol remarks, "nevertheless, the arsenious acid employed at Stockholm by Arrel, in Prussia by Metsger, in England by Bell, and in France by Desgranges, of Lyons—never effected a single cure of cancer, and frequently occasioned casualties requiring a discontinuance of its use." Cayol never saw any bad effect result from arsenious acid, or from the arseniate of soda used by him in treating ague, "according to the formula published lately by M. Foderé."

*Carbonate, Muriate, and Tartrate of Iron.*

Experiments, &c. and recorded results are too few to justify any deductions from the employment of the above preparations.

*Muriate of Baryta.*

Though confidently proposed by Crawfurd, of London, has enjoyed but an ephemeral reputation. Of the three cases (only!) given by Crawfurd, Cayol admits one only to have been truly cancerous. Pinel and Alibert found that water saturated with muriate of Baryta as recommended by Crawfurd, induced casualties, when carried beyond six drops per dose.

We conclude this article with an epitome of the instructive contents of the remaining portion of the chapter entitled—

DIFFERENT EMPIRICAL RECIPES.

*Grey Lizard.*—(Lacerta Agilis.) Is one of the *anti-cancerous* remedies, the virtues of which have been the more exalted on account of its singularity. J. Flores, of the University of Guatimala, in Mexico, was the first who published on the subject in 1781, his memoir being reprinted the year following, at Madrid.

His recipe ran thus—the heads and tails of lizards to be cut off, and after stripping off their skins and entrails, to be bolted forthwith, "tout palpitans." One, two, or three, were thus to be devoured daily. Cataplasms were applied to the cancers. The use of this specific was said to be followed with febrile heat, anxiety, sweats, profuse alvine and renal discharges—convulsions, &c. The grey lizard was so celebrated by the Spanish physicians, that Daubenton and Mauduyt, two celebrated naturalists, were requested by the Royal Academy to ascertain its species. The case of a wench at Cadiz was much spoken of, who got cured in the space of twenty days of an ulcered cancer of the breast, by gobbling a lizard every morning. Theses were published on the virtues of the lizard in Sicily and Germany by Grass and Roëmer. It appears that the French Doctors were not so successful in *their* experiments. M. Bayle directed a man affected with a cancerous tumor of the face to take the remedy—fifty grey lizards were swallowed in the space of fifteen days! "this remedy so vaunted produced no physiologic nor therapeutic effect."

*Bleeding.*

Repeated blood-letting has been included among the curative means of cancer by many authors, more particularly by Valsalva, and Fearon, of London. By Fearon, leechings and topical applications of lead were ordered. If the cancer were internal, general blood-letting was practised, and with effect much more gratifying than could be brought about by opium or hemlock. Milk and vegetable diet only was permitted.

*Pure Water.*

Nothing, not even pure water, has been excepted as calculated for curing cancer, or preventing recurrence after an operation. Pouteau, of Lyons, flattered himself, that many had been radically cured by drinking five or six pints of iced water, during the four and twenty hours, *as the sole nourishment.* The appetite ceased in the course of the third day, his patients bearing the privation well. "There was one who lived fifty days, and even two months without tasting any thing but pure water." If the breath became sour, and the tongue lemon-coloured, two or three drachms of magnesia were given in the forenoon. In the course of two months the patients returned cautiously to the use of soups and solid aliment. Cayol says that, after examining Pouteau's facts, "it will be seen, that he cured nothing more than chronic phlegmasies." Dr. Lamb adopting the ideas of Pouteau, recommended patients affected with cancer to be nourished with distilled water only, for a certain time.

The *examen* of the different treatment recommended up to this hour is not pushed further by Cayol. Of the numerous pretended specifics, there are few to be found which can be employed with benefit, not in curing, but even in assuaging pain for a transient period. "When we consider all that has been said and published upon this subject by trustworthy men (for we do not here speak of vagabonds and charlatans)—it were devoutly to be wished for the honour of medicine, that we could attribute so many falsehoods to the nature of the disease and the difficulty of diagnosis. But, independently of these causes, we cannot overlook the illusions of self-love, the desire of notoriety which speculates on the dearest interests of humanity, and a shameful weakness, not permitting fruitless attempts to be avowed, and which leads so frequently to imposture: a kind of moral degradation, the *tableau* of which is not less afflicting than that of our physical infirmities!

Although the public have so frequently been abused, we must avoid concluding, that remedies which may yet be proposed for the treatment of a disease as yet regarded incurable, should be rejected unexamined. The nature of syphilis is not better known than that of cancer, and it is infinitely more varied in its forms; nevertheless, we are acquainted with a mean of cure, and the mean is purely empirical: it was by a suite of experiments and trials that the discovery was attained; and why may not a specific against cancerous diseases be also discovered?

Assuredly praise is due to those who devote themselves to experimental researches on the treatment of maladies reputedly incurable; but such attempts should be directed with prudence, and the results candidly published."

---

### VAGINAL DISCHARGE IN VERY YOUNG FEMALES—SUSPECTED VIOLATION.

A girl, seven years of age was brought by her mother to Dupuytren's consultation for his advice respecting what she thought were the effects of violence committed on her child. There was a copious yellow-coloured discharge from the vagina, and the labia were red, swollen, and painful. No excoriation or laceration however was to be perceived, and the hymen remained perfect. Dupuytren assured the parents that the symptoms by no means justified the suspicion which they entertained; he could not say positively that no attempts had been made to injure the child; but only that the existing symptoms might arise from other causes. Indeed so frequent are cases of this description at particular periods that some have suspected that they may depend upon some epidemic influence.

Dupuytren was lately consulted by a lady about her young daughter, in whom a purulent vaginal discharge coming on without any apparent cause had been observed for several days; it was of a greenish yellow colour, stained the linen deeply, and was so acrid as to occasion painful micturition. Dupuytren regarded the case as one of catarrhal inflammation of the genitals; and predicted at the time that in all probability several cases of a similar nature would present themselves to his notice in the course of the week: and so it was: they were all treated successfully with tepid baths, demulcents and soothing washes.

In the record of the Faculty of Medecine for 1815, there is the report of a commission (consisting of MM. Dupuytren, Roux, Dubois, and Desormeaux) on a case of suspected violation on the body of an infant only 14 months old, in consequence of a statement which Dr. G——d had made to the prefect of the police, "that the hymen of the infant had been torn, and that the laceration was quite recent." We may here state that the mere circumstance of the hymen being entire, or not, is far from being a conclusively exculpatory or condemnatory circumstance; it may be entire, and yet the legal crime of rape may have been perpetrated; and on the other hand, it may be destroyed in other ways from that of sexual pollution. Moreover we have already seen that the existence of a discharge from the vagina is most unsatisfactory evidence; and the same remark may be made as to the occurrence of tumefaction and even of ecchymosis of the external genitals: in short, these and other symptoms merely indicate the presence of an inflammation, but do not at all point to the cause of that inflammation, and we must be careful not at once to suspect a crime, merely because it is possible that it may have been committed. The commission above named acted judiciously in limiting their report to the bare statement, that the infant was affected with a puriform discharge.—*Journal Hebdom.*

---

### ON CERTAIN MENTAL ILLUSIONS.

In a memoir read by M. Esquirol before the Institute, in October, 1832, the learned author endeavoured to establish the distinction between mental hallucinations and mental illusions: the former are the mere offspring of the disturbed mind, arising without any evident cause, and referable to no distinct operation preceding them; whereas the latter are the results of certain impressions made upon the body, but the nature of which impressions the insane strangely mistakes and perverts: the former are altogether mental phenomena; the latter are mental indeed, but generated from and depending upon previous corporeal influences. These corporeal influences are very generally painful sensations, either on the more immediate organs of sense, or on some of the viscera contained within the great cavities. A few examples will readily indicate that set of cases which Esquirol denominates "illusions."

*Illusions depending upon a Disturbance of the Organic Sensibility of the Encephalon.*

*Case* 1. A young lady, 18 years of age, having for some time suffered from a severe pain on the crown of the head, at length insisted that there was a worm within her head devouring her brain. No reasoning could disabuse her mind of this illusion; and she was convinced that the only method of relieving her was the extraction of the worm. An incision was accordingly made through the scalp, and a portion of fibrine was drawn out, with much ceremony, as if to corroborate the truth of her prediction. She was satisfied, and expressed great pleasure. The wound was kept open for two or three months, by which time, not only all the pain, but the mental illusion as well, had entirely subsided.

*Case* 2. A country woman was admitted into the Salpetrière, in consequence of intense pain at the top of the head, which she attributed to the presence of some animal concealed under the skin. An incision was made through the scalp at the pained spot, and a portion of an earthworm was then shewn to the patient, as if it had been extracted from the wound. She testified great joy at the time, being assured, she said, of her speedy recovery; but the hope of this fortunate event was soon baffled, for, in a fit of rage, she tore open the issue which had been established at the wound, and the mind became as unhinged as ever.

*Case* 3. A lady, 30 years of age, who had fallen into a state of hypochondriasis, after suffering great mental distress, was impressed with the idea that her brain had become petrified. Some time after, she thought that it had softened, and was in a fluid state. On the dissection of this patient, a scrofulous deposit was found in the anterior lobe of the cerebrum.

*Illusions depending upon a Disturbance of the Organic Sensibility of the Abdominal Viscera.*

*Case* 1. Ambrose Paré mentions the case of a hypochondriac, who believed that he had some frogs in his stomach, and who was cured by a smart purgative or two, the attendants having cunningly introduced a frog or two into the night-stool before the patient went to it.

*Case* 2. M. Esquirol examined the body of a woman who had died at the "Salpetrière;" she had believed that some cruel animal was in her stomach. This viscus, at its pyloric orifice, was found cancerous.

*Case* 3. In 1832, there was a woman in the Salpetrière, who suffered from repeated attacks of severe colic. Her own idea was, that a regiment of soldiers lay concealed in her belly, and that she could feel them struggling and fighting with each other. Dissection shewed that a chronic peritonitis had existed for a length of time, and had glued large portions of the intestines together.

*Case* 4. A woman, 57 years of age, of a strong and sanguineous temperament, and so extremely devout and religious in her way, that she was styled by her keepers "la mère de l'église," because she was constantly talking of some scriptural subjects, was impressed with the belief, that all the apostles and evangelists had taken up their residence within her bowels; moreover, that the Pope held his conclave there; and that, occasionally, even some of the Patriarchs of the Old Testament sojourned with his holiness. On examining the abdominal viscera of this woman after death, it was discovered that all the intestines had become agglutinated together, in consequence of chronic peritonitis.

*Case* 5. Similar morbid appearances were found in the abdomen of a lunatic, who believed that a devil had taken up his abode in his stomach to torture him. In this case, the skin, over almost every part of the body, was singularly insensible to mechanical injury; it might be pricked, pinched, and cut, without the patient appearing to suffer any pain.

*Case* 6. A lady, aged 40, mother of several children, had enjoyed good health up to the year 1826, when she experienced a dreadful shock to her feelings, from seeing a young girl of her acquaintance killed by a carriage being driven over her body; she became affected with violent palpitations, with syncope and hæmoptysis; and being summoned to give her evidence upon the trial, other symptoms, such as vomitings, convulsions, grinding with the teeth, a sensation as if something was alive in her stomach, and every now and then was rising up her throat, began to shew themselves. This delusion became gradually more and more fixed in her mind; and no reasoning could dissuade her from the belief, that a common earthworm had found its way into her bowels, and was gnawing and eating them away. This lady was living at the time in the country, and a solemn consultation was held among the wise women of the village, respecting what should be done to expel this worm; at length, the ingenious idea occurred to them, that this worm, if actually in the stomach, might be fished out with a piece of bait, on the very best and most acknowledged rules of angling! For this purpose, the heart of a pigeon was taken and made fast to one end of a string; it was then swallowed by the patient, and after having remained for a sufficient length of time, to entice the worm to lay hold of it, it was withdrawn by means of the other end of the string! but, alas! neither worm, nor even pigeon's heart returned upwards; for the monster had been so greedy as to bite through the string just above where it was secured round the bait!! Some of the savantes proposed to repeat the operation, affixing a hook at the end of the string, which was to be introduced into the stomach; but their advice was over-ruled, and nothing further was done; as her friends now insisted that some regular physicians should see her.

At first her malady was considered as simple hypochondriasis, dependent probably upon vermination, and Dupuytren among the number being consulted, wrote a prescription for a mixture consisting of jalap, pomegranate bark, and calomel; but her husband, (who seems to have been not much more sane than his lady,) upon seeing the word calomel, tore the prescription in pieces, vowing that such a remedy was given only to libertines. All the doctors of Paris were consulted in their turns; some would do nothing; others advised the use of different anthelmintics. M. Manry, physician of the hospital St. Louis, regarding the case as one of pure delusion, thought that it might be most easily cured by a counter-delusion; and therefore, seeming to agree with the patient's opinion of some live animal being in her stomach, he proposed to extract it by an operation. Accordingly, an incision was made through a fold of the integuments of the epigastrium, (as in making a seton,) and the patient made to believe that the stomach was now laid fairly open, and that a blind-worm could be distinctly seen lying at one corner of it; that with some difficulty it was seized and drawn outwards till the patient could lay hold of it herself, and gradually extracted it. All the good anticipated was not however obtained in this instance; for most unfortunately the patient began to suppose that another worm had taken up its abode since the expulsion of the former one, and so quick of growth was this second worm imagined to be, that in the course of a few days it had reached the length of 200 ells.

The issue of this case is not known, as the woman returned into the country.

In the following case, the deception practised was much more successful. A young countryman was fully persuaded in his own mind that he had swallowed a serpent when drinking some water that was rather muddy; that it had gradually grown larger and larger; that it moved about in all directions; sometimes up into his head, at other times down to his anus; now encircling the heart, and now twisting itself round his bladder. When it was disposed to be rather too lively in its motions, the patient said he was obliged to seize hold of one of its rings, through his abdominal parietes, and squeeze it firmly. He repeatedly tried to kill it, by pushing a long sharp needle into its head, but the instrument has always slipped off when he made the attempt. Whenever he was hungry, the serpent always became importunate. On all other subjects this patient was quite rational; and as he urgently requested that this serpent should be extracted, because he was assured that he should be quite well then, M. Manry performed the operation, as in the preceding case; and from that hour all his sufferings vanished. One day indeed he began to be alarmed at some uneasy feelings in the stomach, which he thought might arise from some ova of the serpent having been left behind; but his fears on this score were at once quieted, when he was told that the serpent was a male. The cure in this case was permanent.

*Illusions depending upon a Disturbance of the Sensibility of the Generative Organs.*

A woman, well advanced in years, was haunted with the idea that the devil had crept into her womb, and that nothing could dislodge him from his dark abode. This woman died, and it was found on dissection that there were several hydatids on the external surface of the uterus.

In another case, in which the patient believed that some venomous animal had entered her womb, and was accordingly in the habit of pushing pieces of meat up her vagina to entice it down—a similar disease was found affecting the uterus.

Such are a few examples illustrative of delusions which appear to take their origin from corporeal impressions, acting upon a disturbed mind. We mean not to enter into any investigation of this most abstruse subject: our only motive has been to afford some authentic data to the enquirer.—*Journ. Hebd.*

---

### Further Particulars respecting the Medicinal Properties of the Creosote.

In a former, as well as in this present number, we have alluded to the general properties of this recently-discovered substance, which from all accounts seems to be a potent, and consequently, under a judicious use, a useful remedy. A drop put upon the tongue causes very severe pain, and blisters the part; even the epidermis is usually detached from a portion of the skin, moistened with the pure creosote.

The solution of it in water is the preparation which Reichenbach has chiefly employed;—its strength may be made to vary according to the strength of the irritation required; but in most cases a solution of one part of the creosote in fifty of warm water will be found most convenient. He has used it successfully in scalds and in burns, whether the epidermis has been detached or not; in numerous cases of chronic herpes and impetigo, in itch, &c. When these cutaneous diseases are very obstinate and resist the effects of the creosote solution, he is in the habit of applying the substance either directly and in its pure state, or mixed with lard, so as to form an ointment;—under the use of this ointment, the pustules or vesicles very quickly dry and fall off. The period usually required for the cure varies from one to three weeks; and as a matter of course, this must depend on the duration of the disease, and on the constitution of the patient. Several cases of troublesome, and sometimes apparently malignant ulceration are reported as having been cured by the creosote. Scrofulous ulcers are benefited by it in an especial degree; and when there are any sinuses or fistulæ, no injection will be found so useful as water impregnated with the creosote. If the ulcers should be obstinate, it may be well to touch the edges of them occasionally with the pure creosote; but in most cases the application of pledgets of linen wet with the solution will be found sufficient. Tooth-ache may be often cured instantaneously by introducing a drop of it into the cavity of the decayed tooth. Even the mere gargling with the solution in water, will not unfrequently relieve the pain. The efficacy of the use of the creosote internally we should deem much more problematical, and especially in such a disease as hæmoptysis, for which it has been recommended; the dose given was four drops rubbed up with lump sugar. (it is not stated whether the four drops are to be given in one or in divided doses,) and this was repeated for six or seven days.—*Bulletin Therapeut.*

---

### HYPERTROPHY OF THE MAMMÆ.

Galen is the earliest author who has noticed this malady, and most of the comprehensive records of disease published since his time present some cases of it. Boulli mentions the case of a woman whom he saw at Koenisberg, and in whom the mammæ had become so prodigiously enlarged that she was obliged to support them with bandages, passed round her body and across her shoulders; each of them must have weighed 30 pounds at least. This poor woman had been advised to have the mammæ extirpated, and this murderous operation would have been performed, had Boulli not happened to visit her; he at once discovered that the hypertrophy of the gland was connected, or at least associated, with amenorrhœa, and ordered emmenagogue medicines, derivatives, leeches to the ankles, cupping-glasses to the hips, &c. The result was quite satisfactory; the menstrual flux was restored, and the volume of the mammæ speedily decreased.

Indeed it is a very common occurrence, that when the catamenia have been suddenly checked, the mammæ became swollen and painful. Dorsten relates the case of a young lady, in whom an extraordinary enlargement of the mammæ took place in the course of one night; it was very evidently connected with the retention of the milk, the lady being a nurse at the time; the left breast measured 37 inches round its base, and 18 inches from the base to the nipple—the right one 31 inches round, and 17 in height. Dorsten employed emollient fomentations and revulsive remedies; but, unfortunately, his patient was so feeble, that she could not continue the course prescribed. The catamenia were suppressed for six months, all the means which had been used to restore them having proved quite ineffectual. The lady died; and when the breasts were weighed after death, the left one was found to weigh 64 pounds. No decided structural change could be detected in the gland, except the mere hypertrophy of the cellular tissue which enters into its composition.

Hey, in his Practical observations on Surgery, alludes to several such cases, in all of which the enlargement of the mammæ was associated with amenorrhœa: one of these is so remarkable that it deserves notice. A young girl, æt. 13, on the first occurrence of the menstrual flow, had imprudently put on a damp chemise, with the hope of stopping it; the discharge was thus suddenly arrested, and could not be recalled: the mammæ forthwith began to swell, and gradually attained such a size, that she could not keep herself erect, but was obliged to bend her head and body to diminish the extreme tension, and to draw her limbs up to her stomach, for the purpose of supporting the huge pendulous glands. The left mamma, being the most cumbersome, was extirpated—it weighed 15 pounds. The girl was cured, but ever afterwards had a slight curvature forwards of the spinal column.

The hypertrophy of the mammæ sometimes takes place during pregnancy, and disappears with the cessation of the milk fever after delivery. When the nipples are too small, so that the child cannot easily take hold of them, the tendency to the engorgement of the glands is necessarily greater. Professor Cerutti alludes to the case of one of his patients, who was in this condition. The left mamma attained the dimensions of 38 inches in circumference, and 15 in height.

This woman was safely delivered, and when the milk fever set in, the breasts did not seem to become any longer; as it subsided, they very rapidly decreased in volume, and in the course of a month or so, they measured round their bases only 18 inches.—*Meckel's Archives fur die Physiologie.*

